# Reductions in behavioral deficits and neuropathology in the R6/2 mouse model of Huntington’s disease following transplantation of bone-marrow-derived mesenchymal stem cells is dependent on passage number

**DOI:** 10.1186/scrt545

**Published:** 2015-02-19

**Authors:** Julien Rossignol, Kyle D Fink, Andrew T Crane, Kendra K Davis, Matthew C Bombard, Steven Clerc, Angela M Bavar, Steven A Lowrance, Cheng Song, Steven Witte, Laurent Lescaudron, Gary L Dunbar

**Affiliations:** Field Neurosciences Laboratory for Restorative Neurology, Brain Research and Integrative Neuroscience Center, Program in Neuroscience, 1280 East Campus Drive, HP Building Room 2336, Mount Pleasant, MI 48859 USA; College of Medicine, Central Michigan University, Mount Pleasant, MI 48859 USA; Faculté des Science et des Techniques, Université de Nantes, 44300 Nantes, France; INSERM U1064, ITUN, 44093 Nantes, France; INSERM U791, Laboratoire d’Ingenierie Osteo-Articulaire et Dentaire (LIOAD), 44042 Nantes, France; Field Neurosciences Institute, Saginaw, MI 48604 USA

## Abstract

**Introduction:**

Huntington’s disease (HD) is an autosomal dominant disorder caused by an expanded CAG repeat (greater than 38) on the short arm of chromosome 4, resulting in loss and dysfunction of neurons in the neostriatum and cortex, leading to cognitive decline, motor dysfunction, and death, typically occurring 15 to 20 years after the onset of motor symptoms. Although an effective treatment for HD has remained elusive, current studies using transplants of bone-marrow-derived mesenchymal stem cells provides considerable promise. This study further investigates the efficacy of these transplants with a focus on comparing how passage number of these cells may affect subsequent efficacy following transplantation.

**Methods:**

In this study, mesenchymal stem cells isolated from the bone-marrow of mice (BM MSCs), were labeled with Hoechst after low (3 to 8) or high (40 to 50) numbers of passages and then transplanted intrastriatally into 5-week-old R6/2 mice, which carries the N-terminal fragment of the human HD gene (145 to 155 repeats) and rapidly develops symptoms analogous to the human form of the disease.

**Results:**

It was observed that the transplanted cells survived and the R6/2 mice displayed significant behavioral and morphological sparing compared to untreated R6/2 mice, with R6/2 mice receiving high passage BM MSCs displaying fewer deficits than those receiving low-passage BM MSCs. These beneficial effects are likely due to trophic support, as an increase in brain derived neurotrophic factor mRNA expression was observed in the striatum following transplantation of BM MSCs.

**Conclusion:**

The results from this study demonstrate that BM MSCs hold significant therapeutic value for HD, and that the amount of time the cells are exposed to *in vitro* culture conditions can alter their efficacy.

## Introduction

Huntington’s disease (HD) is an autosomal dominant disorder caused by an expanded and unstable CAG trinucleotide repeat that results in a progressive degeneration of neurons, primarily in the putamen, caudate nucleus, and cerebral cortex. The underlying pathology of HD is initiated when the gene that codes for the huntingtin (htt) protein, located on the short arm of chromosome 4, contains an increased number of CAG repeats [1]. HD in adults is characterized by cognitive impairment and psychiatric disturbances, such as irritability, aggressiveness, and depression, which precede involuntary motor disturbances [1, 2], with death usually occurring 15 to 20 years later.

The R6/2 mouse model of HD expresses the N-terminal portion of human htt, containing a highly expanded glutamine repeat (145 to 155). These mice develop progressive neurological phenotypes resembling HD [3]. At birth, R6/2 mice are indistinguishable from wild-type (WT) littermates and develop normally until 6 to 8 weeks of age, when they begin to express the HD phenotype, initially consisting of neurological signs of stereotypical hind-limb grooming, dyskinesia, irregular gait, and motor dysfunction [4, 5]. The R6/2 model also displays significant reductions in brain-derived neurotrophic factor (BDNF), a protein necessary for striatal neuron survival that is reduced in HD patients [6, 7]. Studies aimed at increasing BDNF within the striatum have shown beneficial results in transgenic animal models of HD, adding evidence to the therapeutic role of BDNF [8–10].

Over the last several decades, stem cell transplantation has gained significant attention as potential treatment for neurodegenerative diseases, including HD, as patients with HD have received clinical benefits from implants of fetal/embryonic stem cells [11–13]. Despite some encouraging results, the use of fetal/embryonic cell sources for therapeutic transplantation is still subject to logistical, immunological, and ethical limitations [12, 14, 15].

To avoid some of these complications, use of adult, bone marrow (BM)-derived stem cells have gained considerable interest. Cultured mesenchymal stem cells (MSCs) are characterized by plastic adherence, rapid proliferation, and multipotency [16]. Transplantation of BM MSCs into the striatum of rodent models of HD has been shown to reduce behavioral deficits [17] and provide neurotrophic support (for a review, see [18]).

Given that MSCs are readily available and can provide functional efficacy following transplantation, they hold considerable promise as a source for an effective cell therapy. However, in order to expand BM MSCs in sufficient numbers for transplantation, *in vitro* passaging, which has shown to alter the properties of the cells [19], is necessary. Our previous work suggested that reducing the number of cell passages may increase transplant survivability in rats and increase their efficacy in reducing behavioral deficits in the 3-nitropropionic acid rat model of HD [20].

The goals of the present experiment were to test the efficacy of BM MSCs in the R6/2 transgenic mouse model of HD, and to determine whether increased passaging of MSCs *in vitro* can alter functional outcomes following transplantation. Behavioral and histological analyses were performed to examine the efficacy of both low-passage (passages 3 to 8) and high-passage (passages 40 to 50) BM MSCs transplanted into the striata of R6/2 mice.

## Methods

### *In vitro*cell characterization

The extraction protocol for obtaining BM MSCs was adapted from previously published procedures [10]. Briefly, BM was aspirated from the fibula of adult (6-week-old to 8-week-old) WT littermates of R6/2 transgenic mice (C57/BL6 background; Jackson Laboratory, Bar Harbor, ME, USA) using a 25 gauge syringe. The cells were then suspended in 10 ml MSC medium (alpha modified Eagle’s medium (Sigma, St Louis, MO, USA) with 10% fetal bovine serum (Invitrogen, Carlsbad, CA, USA), 10% horse serum (Invitrogen), and 5 mg/ml streptomycin and 5 UI/ml penicillin (Sigma)). Following incubation for 48 hours at 37°C, the medium was replaced with fresh MSC medium to remove nonadherent cells.

When cells reached 85% confluency, the cells were passaged. Briefly, the culture medium was aspirated, 0.25% trypsin/ethylenediaminetetraacetic acid solution (Sigma) was added for 5 minutes to detach the cells, and trypsin was then deactivated with 2 ml fetal bovine serum. The trypsin/ethylenediaminetetraacetic acid solution and fetal bovine serum containing the cells was collected and centrifuged at 1,500 rpm for 7 minutes at 4°C. The supernatant was removed and the pellet was then suspended, resuspended, counted, and replated at a density of 8,000 cells/cm^2^ in a new 75 cm^2^ flask (Phenix, Candler, NC, USA) with fresh MSC media. At low passages, 85% confluency of the MSCs was reached initially at every other week and, subsequently, once each week. When growth kinetics stabilized, around passage 10, MSCs reached 85% confluency twice per week.

At passage 6 (low passage) and at passage 50 (high passage), the BM MSCs were analyzed using immunocytochemistry. Briefly, BM MSCs were plated into six-well plates containing poly-l-ornithine coated glass coverslips (25 mm #1; Thermo Fisher Scientific Inc, Waltham, MA, USA) and cultured in MSC medium. At 80% confluency, the cells were fixed with 4% paraformaldehyde in 0.1 M phosphate-buffered saline (PBS) at 4°C for 10 minutes. To block nonspecific binding sites, the coverslips were incubated for 1 hour at room temperature in PBS with 0.1% Triton-X (Sigma) and 10% normal goat serum (Sigma). Following blocking, the coverslips were incubated in primary antibodies overnight at 4°C. Primary antibodies included CD45 (1/500; Abcam Cambridge, MA, USA) and SCA1 (1/500; Abcam). After 24 hours, the coverslips were then rinsed and incubated for 1 hour at room temperature with the appropriately conjugated secondary antibodies. The secondary antibodies included AlexaFluor488 and AlexaFluor594 (1/300; Invitrogen). The coverslips were then rinsed and incubated in Hoechst 33358 (1/1,000; Thermo Scientific Thermo Fisher Scientific Inc, Waltham, MA, USA) for 5 minutes at room temperature to visualize cell DNA and then mounted onto glass slides using Fluoromount (Sigma). Slides were imaged at 20x using a Zeiss Axiovert 200 M inverted fluorescent microscope (Zeiss, Thornwood, NY, USA).

The low-passage and high-passage BM MSCs were analyzed by flow cytometry at passages 8 and 45, respectively, following protocols published previously [21]. Briefly, 200,000 to 300,000 cells were plated into each well of a round-bottom 96-well plate following a passage. The cells were rinsed in 0.1 M PBS containing 1% bovine serum albumin (Sigma) and 0.1% azide (Sigma) and were centrifuged at 2,500 rpm for 1 minute at 4°C. The cells were then resuspended in 30 μl primary antibodies for 1 hour at 4°C. The primary antibodies and dilutions were described above for immunocytochemistry with the addition of the stage-specific embryonic antigen SSEA4 (a marker of stem cells, 1/500; Abcam), major histocompatibility complex (MHC) class I (a receptor for T-cell identification, 1/500; Abcam), and MHC class II (a marker of antigen-presenting cells and lymphocytes). The cells were then rinsed twice and incubated in the secondary antibody,AlexaFluor488, (1/300; Invitrogen, Grand Island, NY, USA) for 1 hour at 4°C. The cells were then rinsed twice and fixed using 4% paraformaldehyde for 10 minutes on ice. The cells were then rinsed and stored at 4°C until analysis was performed using the LSR II (BD Bioscience, San Jose, CA, USA).

Cell cultures were also analyzed for BDNF mRNA expression. Briefly, two million cells were isolated following passaging and stored at −80°C in 200 μl Trizol overnight. RNA isolation was then performed using a Qiagen RNeasy system (Qiagen, Gaithersburg, MD, USA). All procedures followed the manufacturers’ guidelines. The purified RNA was analyzed using a NanoDrop2000 spectrophotometer (ThermoScientific Thermo Fisher Scientific Inc, Waltham, MA, USA), and was then stored at −20°C.

A QuantiTect Reverse Transcription Kit (Qiagen) was used for cDNA synthesis following the manufacturers’ guidelines. Primers used for quantitative PCR were BDNF, tyrosine receptor kinase type 2 (TrkB), tumor necrosis factor alpha (TNFα) and nerve growth factor (NGF). All values were normalized to the housekeeping gene glyceraldehyde 3-phosphate dehydrogenase and the fold increase was calculated from control cDNA isolated from mouse tail-tip fibroblasts. Sequences are located in Table 1.

**Table 1:** **Primer sequences used in quantitative RT-PCR analysis**

Primer	Sequence
GAPDH forward	AAG AGA GGC CCT ATC CCA A
GAPDH reverse	CAG CGA ACT TTA TTG ATG GTA
BDNF forward	GAA GAG CTG CTG GAT GAG GAC
BDNF reverse	TTC AGT TGG CCT TTT GAT ACC
TrkB forward	CTC AAG TTG GCG AGA CAT TCC A
TrkB reverse	AAT CCA GGC ACT TCC TCG TTC
NGF forward	TAG CGT AAT GTC CAT GTT GT
NGF reverse	CCC ACA CAC TGA CAC TGT CA
TNFα forward	GTT CGG ATC CCA CTG TGA CT
TNFα reverse	GTC CCC AGA GCC AAT GAC TA

BDNF, brain-derived neurotrophic factor; GAPDH, glyceraldehyde 3-phosphate dehydrogenase; NGF, nerve growth factor; TNFα, tumor necrosis factor alpha; TrkB, tyrosine receptor kinase type 2.

### Animals

All procedures mentioned were reviewed and accepted under the guidelines of the Institutional Animal Care and Use Committee of Central Michigan University. Male and female R6/2 and WT mice were housed at 22°C under a 12-hour light/12-hour dark reverse light cycle with *ad libitum* access to food and water. The mice were genotyped at 3 weeks of age by PCR and separated into the following four groups balanced by gender and genotype: sham-operated (injection into the striatum of Hanks balanced salt solution; Gibco, Thermo Fisher Scientific Inc, Waltham, MA, USA) WT mice (WT group; *n* = 19); sham-operated R6/2 mice (R6/2 group; *n* = 10); R6/2 mice transplanted with low-passage BM MSCs (R6/2 BM Low group; *n* = 7); and R6/2 mice transplanted with high-passage BM MSCs (R6/2 BM High group; *n* = 10). Following group assignment, the experimenters were blinded to the group identity until the conclusion of the experiment.

### Bone marrow mesenchymal stem cell transplantation

At 5 weeks of age, mice were anesthetized with isoflurane gas and oxygen. The head of each mouse was shaved and cleaned using chlorhexadine (Molnlycke Healthcare Norcross, Georgia, USA). Prior to placing the mouse in the stereotaxic device, 2% lidocaine gel (Hi-tech Pharmacal was just acquired by Akorn, Lake Forest, IL, USA) was placed on the tip of the ear bars. Anesthesia was maintained with isoflurane gas and oxygen for the duration of the surgery. A midline incision was made on the scalp and the skin was retracted, exposing bregma. Two burr holes (0.5 mm) were placed directly over the neostriatum (coordinates relative to bregma: anterior +0.5 mm; lateral ± 1.75 mm; tooth bar set at −3.3 mm). Prior to transplantation, MSCs at either low passage (passages 3 to 8) or high passage (passages 40 to 50) were pre-labeled with Hoechst 33358 (5 μg/ml; Sigma) and resuspended at a density of 200,000 cells/μl in Hanks balanced salt solution. The cells were loaded into a 10 μl Hamilton microsyringe and bilaterally transplanted (2.5 mm ventral to dura) at a constant rate of 0.33 μl/minute for 3 minutes. Following the first injection, the syringe was left in place for 3 minutes, raised 1 mm, and injected a second time (two injections of 200,000 cells). After a second 3-minute wait period, the microsyringe was withdrawn at a steady rate over a 3-minute period. The same procedure was then followed on the opposite hemisphere, the burr holes were sealed with bone wax, and the wound was closed using sterile wound clips (7 mm). The mice were placed in a recovery cage until fully mobile, at which point they were returned to their home cage.

### Behavioral tests

All mice were tested for baseline behavior at 5 weeks of age, prior to cell transplantation. Mice underwent 6 weeks of testing on all behavioral tasks, beginning the week following surgery; except for the Morris water maze (MWM) task, for which testing began 2 weeks following surgery and lasted 5 weeks.

### Rotarod test

The rotarod (SDI Rotor-Rod; San Diego Instruments San Diego, CA, USA) was used to assess motor coordination. The mice were required to maintain balance on a 3 cm diameter rotating rod for 60 seconds. The rod rotated at a constant speed of 10 rpm and each mouse was given three trials per day. If the mice were incapable of remaining on the rotarod for the full 60 seconds, they fell onto a foam pad placed below the apparatus.

### Clasping

The mice also had their limb-clasping response recorded. Procedures followed previously published protocols [8]. Briefly, the mice were suspended by their tails from a height of 50 cm for 30 seconds, and a limb-clasping response was defined as the withdrawal of any limb to the torso for more than 1 second. Each testing session consisted of three trials with a clasping score ranging from 0 to 4, with 0 representing the absence of clasping, 1 representing a withdrawal of any single limb, 2 representing the withdrawal of any two limbs, 3 representing the withdrawal of any three limbs, and 4 representing the withdrawal of all four limbs. The limb-clasping response scores were averaged for each testing session for each animal.

### Morris water maze

An adapted MWM was used to test cognitive function through spatial memory [22]. Briefly, the MWM is a tank of 142 cm diameter filled with opaque water (30.5 cm deep water mixed with nontoxic white paint). A 14-cm diameter platform was placed 1 cm below the surface of the water. Extra-maze visual cues included the testing room door and window on the wall of the south starting location. Prior to baseline testing, each mouse was given a cued trial, where the platform was placed in the center of the MWM with an attached visible flag as a cue. The mice were given four training trials from four starting locations (north, south, east, and west) to acclimatize the mice to the water and the escape paradigm, as well as assessing their visual acuity and swimming ability. During each testing week, the mice were placed in the water facing the south location and the hidden platform was subsequently altered each testing day between the northwest and northeast quadrants, with the platform in the center of the quadrant. During baseline, and on subsequent testing days, the mice were given five trials to find the hidden platform. Following a successful trial, the mice were left on the platform for 5 seconds, removed from the tank, dried, and given a 45 second inter-trial interval. Mice who did not locate the hidden platform within 60 seconds were guided by hand to the platform and allowed to rest on the platform for 5 seconds. The swim speed, distance traveled, and latency to find the hidden platform was measured using Viewpoint VideoTrack version 1.75 (Videotrack; View Point Life Science, Montréal, QC, Canada). However, given the progressive motoric deficits that emerge in the R6/2 mice, the dependent measure used in this study was the percentage of correct responses. Each trial was scored as correct if the mouse was able to find the hidden platform in less than 60 seconds and the percent of correct responses was calculated at the end of each testing session:


### Histological analysis

At the conclusion of behavioral testing, when mice were 12.5 weeks old, one-half of the animals from each group were deeply anesthetized with an overdose of sodium pentobarbital (intraperitoneally), and transcardially perfused with 0.1 M PBS, followed by 4% paraformaldehyde (diluted in 0.1 M PBS at pH 7.4) to fix the brains. The brains were then rapidly removed, suspended in 4% paraformaldehyde for 24 hours at 4°C, and then transferred to 30% sucrose in 0.1 M PBS for 48 hours at 4°C. The brains were then flash frozen using methylbutane (Sigma) and were stored at −80°C until they were processed.

Tissue was sectioned on a cryostat (Vibrotome UltraPro 5000; Leica, Buffalo Grove, IL, USA) at 30 μm, labeled using previously established free-floating fluorescent staining protocols [20] and mounted on positive-charged microscope slides (Globe Scientific Inc., Paramus, NJ, USA). For immunohistochemical analysis, the tissue was labeled with antibodies for neuronal nuclei (mouse NeuN, 1/500; Abcam) and glial fibrillary acid protein (rabbit GFAP, 1/500; Abcam). The tissue was first blocked using 10% normal goat serum in PBS with 0.1% Triton-X for 45 minutes at room temperature. The tissue was then transferred to a well containing the primary antibodies and stored at 4°C overnight. The following day, the tissue was rinsed three times in PBS with 0.1% Triton-X and transferred to a well containing the appropriately conjugated secondary antibodies for 1 hour at room temperature. Secondary antibodies consisted of anti-rabbit AlexaFluor488 and anti-mouse AlexaFluor594 (1/300; Invitrogen).

Cytochrome oxidase (CYO) histology was used to determine the extent of metabolically active tissue, and these same sections were used for subsequent morphological analysis. Briefly, the tissue was submersed in a solution of 800 mg sucrose, 4 mg cytochrome C, and 1 mg diaminobenzadine dissolved in 20 ml phosphate buffer for 4 hours at room temperature on an agitator. The tissue was then transferred to deionized water, mounted onto positively charged glass slides, and coverslipped using Depex (Electron Microscopy, Hatfield, PA, USA).

### Image acquisition and analysis

Images of the fluorescent labels were captured using a Zeiss Axiovert 200 M inverted fluorescent microscope with optical dissectors at 20× magnification. CYO labeled tissue was scanned using Nikon ScanPro (Nikon, Melville, NY, USA). Images were captured from five levels, centered at 0.5 mm anterior to the bregma (the transplant site) with two additional sections anterior and posterior to the transplant site, approximately 200 μm apart.

All images were analyzed using ImageJ (National Institutes of Health, Bethesda, MD, USA). The average intensity of the fluorescent label, the counts of positively labeled cells, as well as the percentage of colocalization between the transplanted MSCs and NeuN or GFAP were analyzed between all groups. The average intensity of the fluorescent label, the number of labeled cells, and the percentage of cells that were co-labeled for both MSCs and either NeuN or GFAP were analyzed from samples of all transplant groups. Methods for rare-event stereology were modified from previously published protocols [23, 24]. Briefly, cell counts were made from the dorsal striatum within each section. Cells were counted as positive cells if they showed: antibody immunoreactivity within the cell body; the nucleus of that cell was within the counting frame without touching the exclusion lines; and the nucleus of that cell was in focus. For cell counts, the average number of cells per section was calculated.

Densitometric measures of CYO and GFAP were taken from the striatum and the average intensities were normalized to the corpus callosum of each section. For the total brain area, sections were traced using ImageJ and the total area was calculated.

### RNA isolation of transplanted brain

Mice that were not used for immunohistological analysis were killed via cervical dislocation when the mice were 12.5 weeks old, and striata of these mice were isolated, flash-frozen with liquid nitrogen, and stored at −80°C. RNA isolation was performed using a Qiagen All-Prep system following the manufacturer’s guidelines. Briefly, striatal tissue was homogenized using a lysis buffer and transferred to an All-Prep spin column and centrifuged at 9500 rpm for 1 minute. Purified RNA was collected in the collection tube and analyzed using a NanoDrop2000 spectrophotometer (ThermoScientific), and was stored at −20°C until used for cDNA synthesis. A QuantiTect Reverse Transcription Kit (Qiagen) was used for cDNA synthesis following the manufacturer’s guidelines. The cDNA was stored at −20°C until use in quantitative PCR experiments. The primers used for gene expression were BDNF, NGF, TrkB, and TNFα. All values were normalized to the housekeeping gene, glyceraldehyde 3-phosphate dehydrogenase, and the fold increase was calculated from control cDNA isolated from mouse brain homogenate. The sequences for each primer are presented in Table 1.

### Statistical analysis

All statistical analyses were performed using SPSS v16 (IBM SPSS Statistics, Armonk, NY, USA) with an alpha level equal to 0.05. Assays between low-passage and high-passage BM MSCs *in vitro* were analyzed using an independent-samples *t* test*.* All behavioral data were analyzed using a repeated-measures analysis of variance (ANOVA), to measure changes between genotypes and treatments across weeks. Histological data were analyzed using a one-way ANOVA. When appropriate, a Tukey’s honestly significant difference (HSD) test was performed for *post hoc* analyses.

## Results

### *In vitro*cell characterization

**Figure 1:**
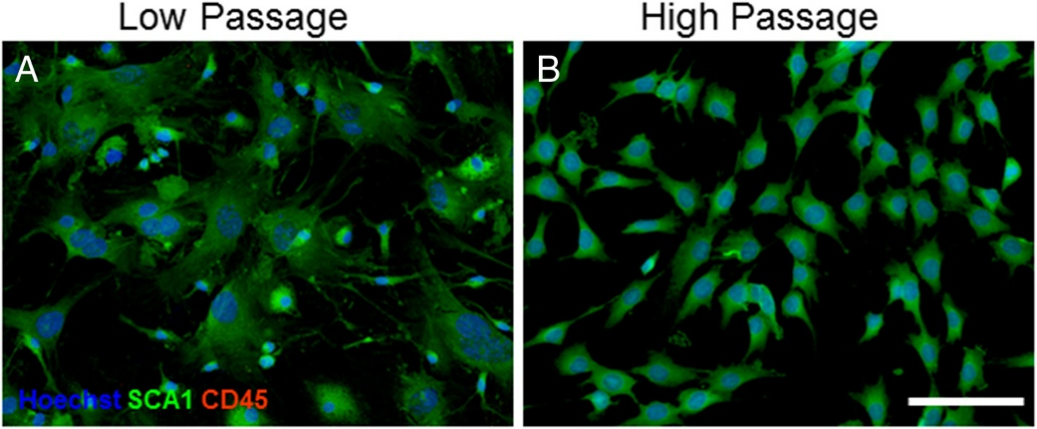
**Immunocytochemistry of low-passage and high-passage bone marrow mesenchymal stem cells . (A)** Low-passage bone marrow mesenchymal stem cells (BM MSCs) and **(B)** high-passage BM MSCs displayed typical MSC morphology, expressed the mesenchymal stem cell marker SCA1, and were negative for the hematopoietic stem cell marker CD45. Scale bar represents 100 μm.

Both low-passage and high-passage BM MSCs showed positive expression of SCA1, a marker of mouse MSCs, without expressing CD45, a marker of hematopoietic stem cells (Figure 1). Flow cytometry analysis (Table 2) revealed that low-passage BM MSCs displayed 14.3% positive expression of SCA1, while high-passage BM MSCs displayed 74.4% positive expression of SCA1, indicating that SCA1 changes over passages. With respect to CD45, analyses of the flow cytometry data revealed a 3.0% and 4.7% expression of BM MSCs at low passage and high passage, respectively. In terms of SSEA4, a marker of stem cells, flow cytometry data indicated an increase from 2.2% in low-passage BM MSCs to 95.5% in high-passage BM MSCs. MHC class I expression remained relatively stable between low-passage and high-passage BM MSCs, at 3.7% and 4.0%, respectively. MHC class II expression also remained relatively stable between low-passage and high-passage BM MSCs, at 2.7% and 4.4%, respectively. These data indicate that either a selection process is taking place over passages, with SCA1^+^/CD45^–^ cells proliferating at a higher rate, or that prolonged exposure to culture conditions causes expression levels to change. In either case, these data suggest that high-passage mouse BM MSCs may prove to be more suitable for transplantation studies.

**Table 2:** **Flow cytometry results of low-passage and high-passage bone marrow mesenchymal stem cells**

	Low passage (%)	High passage (%)
SCA1	14.3	74.4
CD45	3.0	4.7
SSEA4	2.2	95.5
MHC class I	3.7	4.0
MHC class II	2.7	4.4

An increase in the number of cells positively expressing SCA1 was observed as the passage number increased. An increase in the stem cell marker SSEA4 was also observed as the cell passage increased. Expression levels of CD45, MHC class I, and MHC class II remained relatively stable as the passage number increased. MHC, major histocompatibility complex.

**Figure 2:**
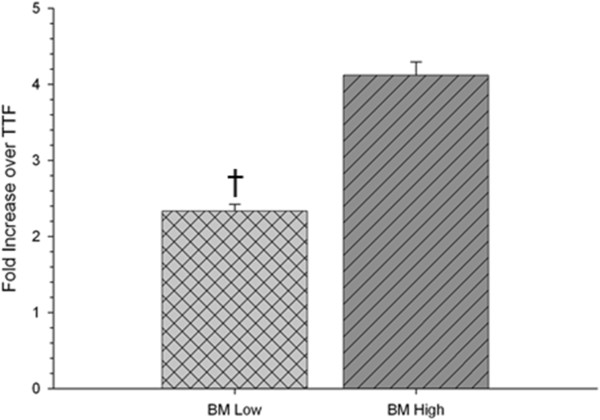
***In vitro*** **quantitative RT-PCR of brain-derived neurotrophic factor mRNA expression of bone marrow mesenchymal stem cells.** A significant increase in mRNA levels of brain-derived neurotrophic factor (BDNF) was observed in the high-passaged bone marrow mesenchymal stem cells (BM MSCs) compared with low-passaged BM MSCs. ^†^Significant from high-passage BM MSCs; tail-tip fibroblasts (TTF) are control cDNA isolated from mice. Bar graph represents mean value; error bars represent standard error of the mean.

An independent *t* test of BDNF gene expression revealed a significant difference between low-passaged and high-passaged BM MSCs (*t*(4) = 9.120, *P* = 0.001), with high passages displaying a significantly higher expression of BDNF mRNA – suggesting that high-passage BM MSCs may prove to be more effective following intra-striatal transplantation, due to greater mRNA expression of the pro-neuron survival trophic factor (Figure 2).

### Behavioral results

**Figure 3:**
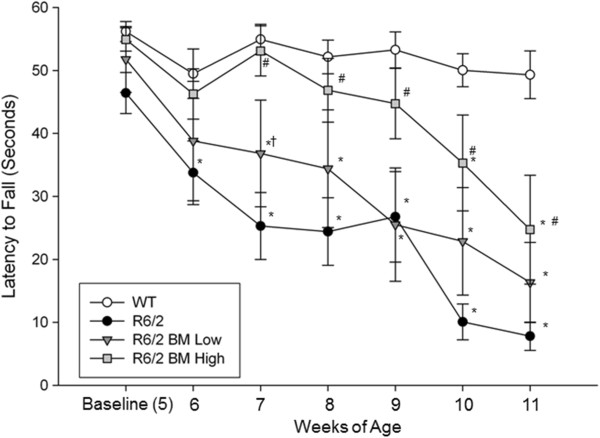
**Assessment of motor coordination in R6/2 mice following bone marrow mesenchymal stem cell transplantation.** A significant decline in motor coordination was observed in untreated R6/2 mice when compared with wild-type (WT) mice. The R6/2 mice who received transplantation of high-passage bone marrow mesenchymal stem cells (BM MSCs) displayed significantly longer latencies to fall compared with untreated R6/2 mice starting at 7 weeks of age and continuing throughout the study. *Significant from WT; ^#^significant from R6/2; ^†^significant from high-passage BM MSCs. Data points on line graph represent mean value; error bars represent standard error of the mean.

In the R6/2 model of HD, clear motoric deficits, tested on the rotarod, become apparent at 6 weeks of age. A repeated-measures ANOVA revealed a significant interaction between weeks and group (*F*(18,174) = 2.247, *P* = 0.004) as well as a significant between-group difference for the latency to fall during the rotarod task (*F*(3,29) = 12.092, *P* = 0.001; Figure 3). A Tukey’s HSD *post hoc* analysis revealed significant motoric deficits between R6/2 and WT mice at each time point following baseline testing. Mice transplanted with low-passage BM MSCs displayed only modest benefits in their performance on the rotarod task, with deficits delayed by 1 week, relative to nontransplanted R6/2 controls.

However, in the R6/2 group that was transplanted with high-passage BM MSCs, the latency to fall from the rotarod was comparable with WT mice starting at baseline (when the mice were 5 weeks old) and continuing until the animals were 10 weeks old. Rotarod performance of mice with high-passage BM MSC transplants were statistically different from nontransplanted R6/2 mice, beginning at 7 weeks of age, with this trend continuing throughout the duration of the study. A significant difference on the rotarod task was also observed between high-passage BM MSC and low-passage BM MSC when the mice were 7 weeks old. Although high-passage BM MSC transplanted mice eventually showed a decline in motor coordination on the rotarod, they still had significantly longer latencies to fall off the rotarod than untreated R6/2 mice, suggesting behavioral sparing occurred, even at the late stages of the progressive HD-like deficits.

**Figure 4:**
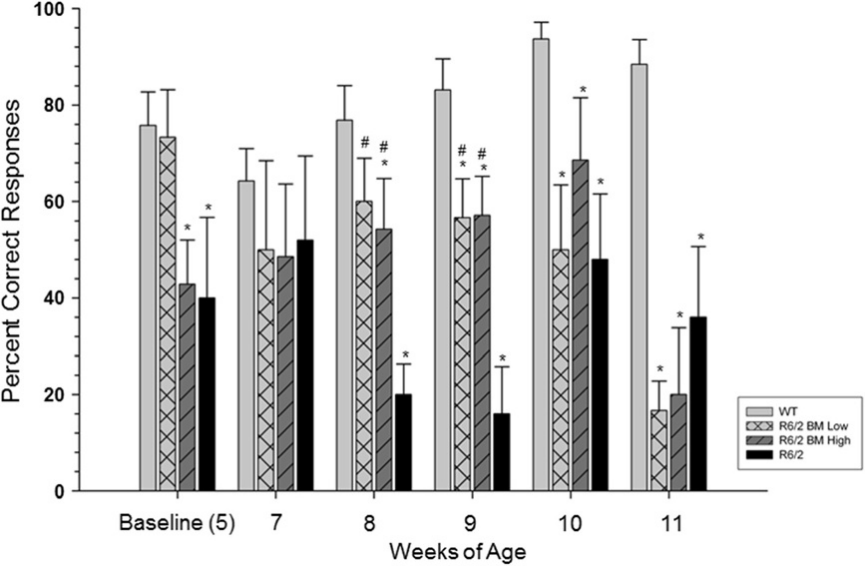
**Assessment of spatial memory in R6/2 mice following bone marrow mesenchymal stem cell transplantation.** Untreated R6/2 mice displayed significant impairment in spatial memory at 5, 8, 9, 10, and 11 weeks of age when compared with wild-type (WT) mice. Mice receiving either low-passage or high-passage bone marrow mesenchymal stem cells (BM MSCs) displayed an intermediate sparing of spatial memory at 8 and 9 weeks of age. *Significant from WT; ^#^significant from R6/2; ^†^significant from high-passage BM MSCs. Bar graph represents mean value; error bars represent standard error of the mean.

Analysis of cognition through MWM testing revealed significant spatial learning deficits in the R6/2 mice, appearing between 5 and 7 weeks of age. A repeated-measures ANOVA revealed significant differences between groups for the percentage of correctly locating the hidden platform (*F*(3,33) = 13.143, *P* = 0.001) and a significant interaction between time and group (*F*(15,165) = 3.014, *P* = 0.001; Figure 4). A Tukey’s HSD *post hoc* analysis revealed significant differences between R6/2 and WT mice during the baseline testing period, as well as significant differences when the mice were 8, 9, 10, and 11 weeks old.

For mice that received transplantation of BM MSCs, an intermediate effect in cognitive performance was observed when the mice were 8 and 9 weeks old, when they had a significantly higher percentage of correct responses than untreated R6/2 mice but still a significantly lower probability to find the hidden platform than WT mice. However, at 9 and 10 weeks of age, both transplant groups showed declines in cognitive performance to the untreated R6/2 level. These data indicate that, while a preservation of cognitive function is possible following transplantation of either low-passage or high-passage BM MSCs, these mice display similar cognitive dysfunction in the later stages of the HD-like progression of cognitive deficits.

**Figure 5:**
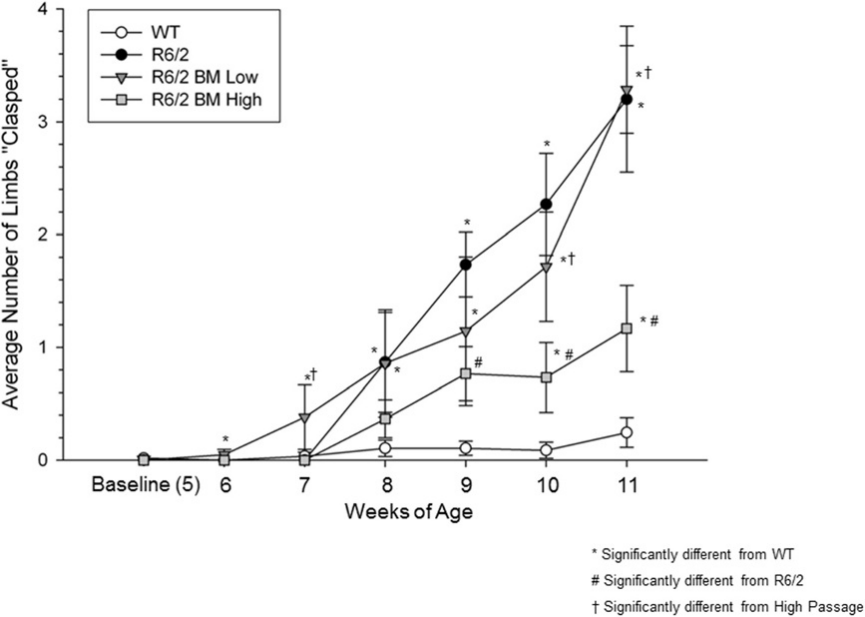
**Limb-clasping response of R6/2 mice following bone marrow mesenchymal stem cell transplantation.** Untreated R6/2 mice had significantly more limb-clasping responses than wild-type (WT) mice starting at 8 weeks of age, with this impairment continuing for the duration of the study. R6/2 mice that received transplantations of high-passage bone marrow mesenchymal stem cells (BM MSCs) had significantly fewer limb-clasping responses than untreated R6/2 mice for the duration of the study, starting at 9 weeks of age. *Significant from WT; ^#^significant from R6/2; ^†^significant from high-passage BM MSCs. Data points on line graph represent mean value; error bars represent standard error of the mean.

The limb-clasping response is a reliable behavioral phenotype observed in many transgenic animals, and may be related to cortical dysfunction in this mouse model of HD [8, 22, 25]. A repeated-measures ANOVA revealed main effect of group in the limb-clasping response task (*F*(3,37) = 20.204, *P* = 0.001), and a significant interaction between week and group (*F*(18,222) = 9.740, *P* = 0.001; Figure 5).

Confirming this phenotypic behavior, Tukey’s HSD *post hoc* analysis revealed significant differences between R6/2 and WT mice beginning at 8 weeks of age and continuing for the duration of the study. A Tukey’s *post hoc* analysis also revealed that R6/2 mice transplanted with high-passage BM MSCs showed a delay in onset of limb clasping until 10 weeks of age, at which point these animals also showed significantly fewer limb clasps compared with R6/2 mice transplanted with low-passage BM MSCs. Significant differences were also observed between the high-passage BM MSC group and untreated R6/2 mice starting at 9 weeks of age. Interestingly, R6/2 mice receiving transplants of low-passage BM MSCs displayed significantly more limbs clasped, compared with WT mice, during all testing weeks, whereas untreated R6/2 mice did not start showing significantly more clasping behavior than WT mice until the mice were 8 weeks old.

### Histological results

**Figure 6:**
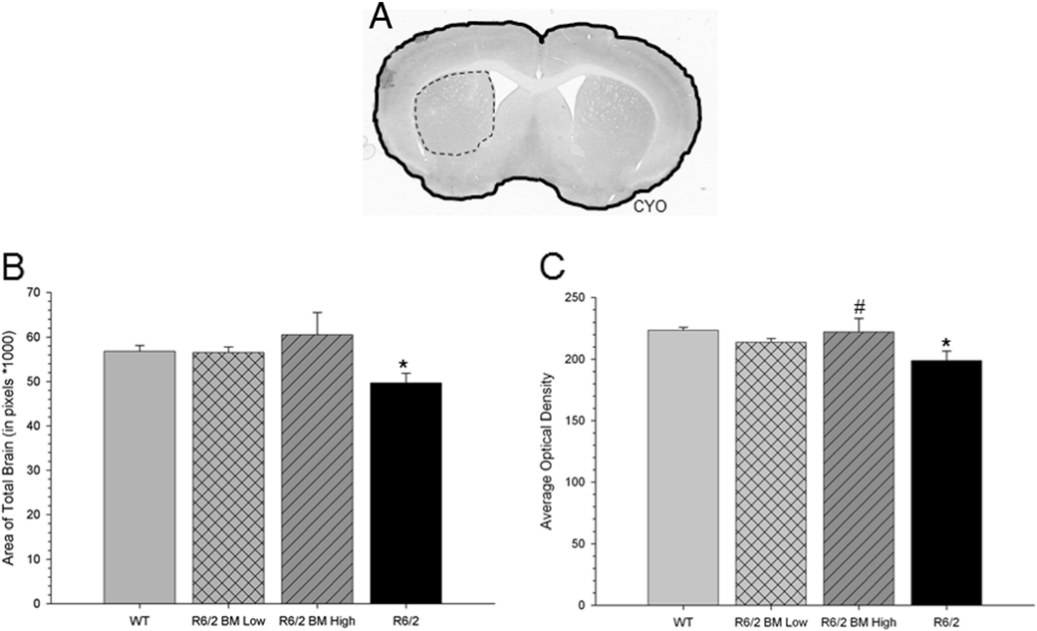
**Measures of brain area and evidence of integrity of the metabolic tissue in the striata of mice receiving bone marrow mesenchymal stem cell transplantations. (A)** Gross morphology of the brain near the area of transplantation can be visualized with cytochrome oxidase (CYO) labeling. **(B)** Untreated R6/2 mice had a significant decrease in total brain area (outlined in solid black) when compared with WT mice. Optical densitometric measures of CYO in the labeled tissue of the striata (outlined in dashed line) revealed significantly less metabolically active area in untreated R6/2 mice when compared with wild-type (WT) mice at the time of necropsy. **(C)** R6/2 mice that received transplantation of high-passage bone marrow mesenchymal stem cells (BM MSCs) had significantly higher levels of CYO labeling in the striata than did untreated R6/2 mice. *Significant from WT; ^#^significant from R6/2. Data points on line graph represent mean value; error bars represent standard error of the mean.

Six weeks following transplantation, when mice were 12.5 weeks old, their brains were extracted, preserved, and analyzed for possible morphological changes, levels of CYO labeling (as a measure of mitochondrial integrity), as well as transplant survival and levels of immune response to the transplanted cells. Scans from five levels of CYO-stained tissue were outlined, and total area of the brain was analyzed for each animal (Figure 6A). A one-way ANOVA revealed significant between-group differences in the area of the whole brain from the sections analyzed (*F*(3,105) = 3.209, *P* = 0.026). A Tukey’s HSD *post hoc* analysis revealed that total brain area in untreated R6/2 mice was significantly smaller than in WT animals, suggesting general brain atrophy. This brain atrophy was not observed in R6/2 mice transplanted with low-passage or high-passage BM MSCs, as both groups were not significantly different from the WT group (Figure 6B).

Optical densitometric analysis of metabolically active tissue in the striatum revealed a between-group difference (*F*(3,105) = 5.896, *P* = 0.002), with untreated R6/2 mice having less density of CYO labeling in the striatum, when compared with WT mice. Mice transplanted with low-passage MSCs were not significantly different from either WT or untreated R6/2 mice. Importantly, the amount of metabolically active, CYO-labeled tissue in R6/2 mice transplanted with high-passage BM MSCs was indistinguishable from those levels observed in WT mice (Figure 6C). These data suggest that transplantation of either low-passage or high-passage BM MSCs can prevent the loss of metabolically active tissue and that transplantation of high-passage significantly reduces the level of metabolic loss when compared with untreated R6/2 mice.

**Figure 7:**
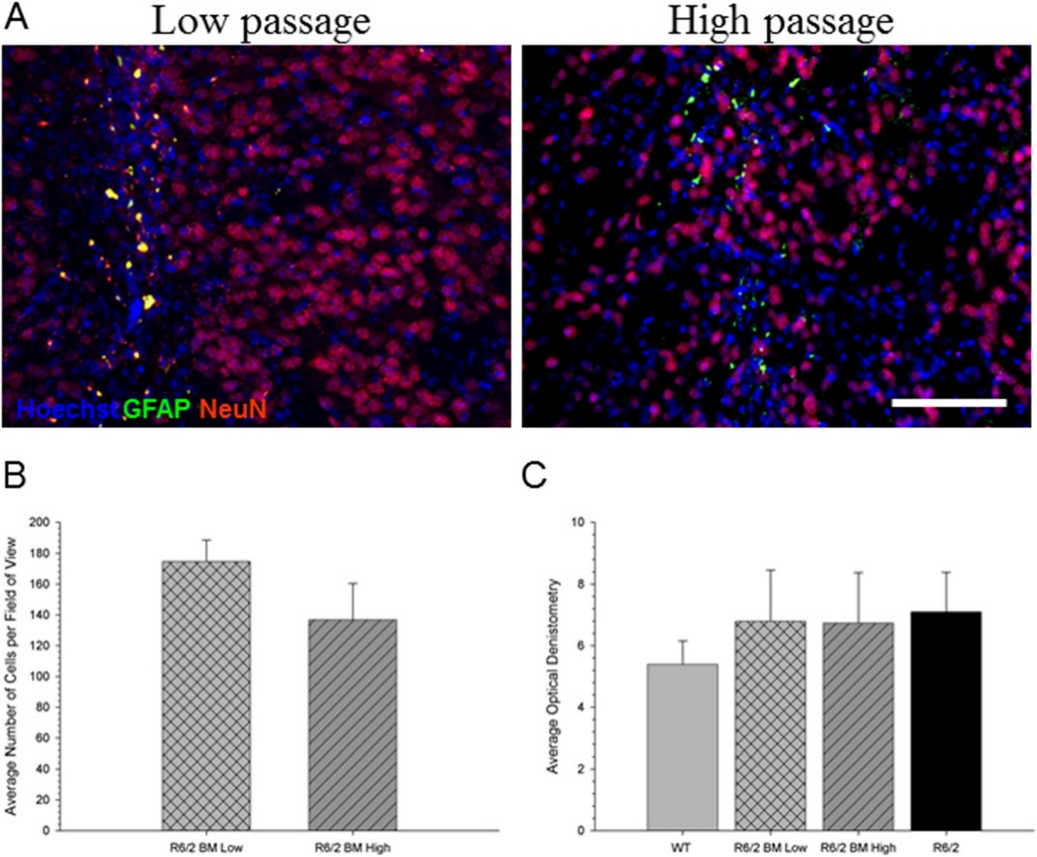
**Immunohistochemical analyses of the transplanted bone marrow mesenchymal stem cells. (A)** No neuronal or glial differentiation was observed from the transplanted bone marrow mesenchymal stem cells (BM MSCs) as seen by the lack of colocalization between the BM MSCs (blue) with neuronal nuclei (NeuN; red) or glial fibrillary acid protein (GFAP; green), respectively in either low-passage (left panel) or high-passage (right panel) BM MSC.**(B)** No significant differences were observed in the amount of surviving BM MSCs between low-passage and high-passage BM MSCs. **(C)** Also, no significant differences were observed between all groups in densitometric measures of GFAP around the transplant site.

Analysis of the number of Hoechst-labeled BM MSCs in the striatum at 6 weeks following transplantation revealed no significant differences between low-passage and high-passage groups (*t*(43) = 1.372, *P* = 0.233), demonstrating that both low-passage and high-passage BM MSCs were capable of surviving in the brain (Figure 7B). To confirm that the Hoechst label was not leaching from the BM MSCs, dead BM MSCs labeled with Hoechst were transplanted into the brain of a normal mouse and there were no positively-labeled cells at 6 weeks following transplantation, confirming that the fluorescent cell linker was not transferred to the host (data not shown). Analysis of images taken from fluorescent microscopy revealed that transplanted cells did not colocalize with immunohistochemistry-labeled tissue for mature neurons (NeuN) and astrocytes (GFAP; Figure 7A), suggesting that BM MSCs did not differentiate into tissue of a neuronal lineage. Optical densitometry analysis of GFAP-labeled tissue from the areas around the transplant site revealed no significant differences between groups (*F*(3,88) = 0.440, *P* = 0.725; Figure 7C).

### mRNA expression

**Figure 8:**
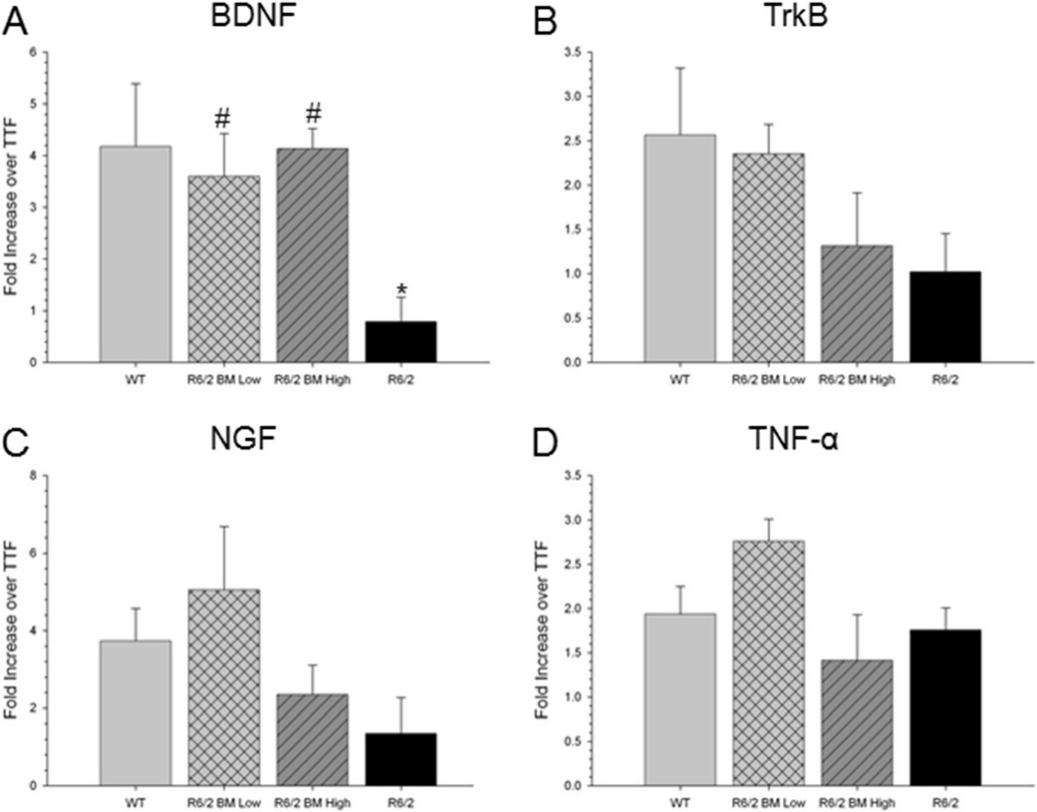
***In vivo*** **quantitative RT-PCR of BDNF, TrkB, NGF, and TNFα mRNA expression (fold increase over tail-tip fibroblast) following transplantation of bone marrow mesenchymal stem cells. (A)** Untreated R6/2 mice had significantly lower expression of brain-derived neurotrophic factor (BDNF) than wild-type (WT) mice, R6/2 mice receiving low-passage bone marrow mesenchymal stem cells (BM MSCs), and R6/2 mice receiving high-passage BM MSCs. No significant differences were observed in the expression levels of **(B)** tyrosine receptor kinase type 2 (TrkB), **(C)** nerve growth factor (NGF), or **(D)** tumor necrosis factor alpha (TNFα) between groups. *Significant from WT; ^#^significant from R6/2. Data points on line graph represent mean value; error bars represent standard error of the mean. TTF, tail-tip fibroblast.

As neurotrophic factors, such as BDNF, play an important role in the pathology of HD, mRNA was isolated from the striatum of mice 6 weeks following transplantation. RT-PCR analysis of BDNF expression revealed a significant main effect of group (*F*(3,15) = 4.134, *P* = 0.032; Figure 8A). A Tukey’s HSD *post hoc* analysis revealed that untreated R6/2 mice had a significant reduction in BDNF expression compared with WT mice. Both BM MSC transplant groups had significantly higher expression of BDNF compared with untreated R6/2 mice. Analysis of expression levels of the BDNF receptor TrkB revealed no significant differences between groups (*F*(3,15) = 1.895, *P* = 0.184; Figure 8B), although a strong trend was observed between WT and R6/2 mice. Similar to TrkB, a one-way ANOVA revealed no significant between-group differences in gene expression of NGF in the tissue dissected from the striata (*F*(3,13) = 2.232, *P* = 0.147), although a trend towards reduced NGF expression was observed in the R6/2 mice compared with WT mice (Figure 8C). Analysis of the proinflammatory gene TNFα revealed no significant between-group differences in gene expression (*F*(3,15) = 2.664, *P* = 0.095; Figure 8D).

## Discussion

This study yielded four main findings: transplantation of BM MSCs into the striata of R6/2 mice significantly delays the progressive motor and cognitive dysfunction associated with this model; the beneficial effects of these cells is dependent on the number of times they have been passaged; transplantation of these cells can preserve anatomical features in the R6/2 brain in the absence of evidence for neuronal differentiation; and transplantation of BM MSCs can prevent/restore the decreased expression of BDNF in the striata of R6/2 mice.

The data from this study corroborate earlier findings from our laboratory demonstrating behavioral and neuropathological sparing in an HD model following transplantation of BM MSCs [8, 10, 17, 23]. This study shows that BM MSCs were capable of slowing the progressive decline in motor and cognitive performance in a highly aggressive mouse model of HD. The current results support our previous work indicating that autologous BM transplants into the striata of rats given intra-striatal injections of quinolinic acid (QA) can reduce cognitive deficits in the radial-arm water-maze task [17]. The precise mechanism whereby this cognitive deficit is ameliorated remains unknown, but may involve the preservation of striatal cells known to be involved with place field properties [26]. The efficacy of MSCs in treating HD-like deficits has also been observed in the QA rat model of HD, using BM MSCs [27]. Other sources of MSCs, such as adipose-derived MSCs/stromal cells, reduced the motor deficits in the R6/2 mouse model [28]. While the suggested mechanisms of action of transplanted MSCs being reported in these studies are different, the overall results of these studies provide converging evidence for the potential use of MSCs as a therapy for HD.

In the current study, we observed *in vitro* changes in gene expression of BDNF, as well as an increase in expression of cell surface proteins, SSEA4 and Sca1, as a result of passaging. Although both low-passage and high-passage MSCs are derived from the same source, it is possible that the process of passaging has selected two separate populations with differential expression of these genes, an effect we have observed previously in MSCs isolated from the umbilical cord [23]. Contrary to what was observed in our previous study using rat BM MSCs, it is apparent that, in this model, mouse BM MSCs are more therapeutically beneficial after extended periods in culture. Given the significance of BDNF in the HD brain [6], we are suggesting that the high-passage mouse BM MSCs were better suited towards delaying the onset of both motor and cognitive deficits in an aggressive mouse model of HD due to significantly higher mRNA expression of BDNF *in vitro*.

Previous results in our laboratory have shown that MSCs decreased their immune response following striatal transplantation [20]. Although the differences in behavioral outcome between transplantation of low-passage and high-passage BM MSCs may have been due to differences in the immunomodulatory effects of these cells, we do not think this is likely, given that the cells did not significantly modulate astrocyte activation following intrastriatal transplantation. For example, expression of a proinflammatory gene (TNFα) and densitometric analysis for immunolabeled reactive astrocytes (GFAP) within striatal tissue did not reveal significant differences between groups. Given the lack of increased inflammation within the R6/2 brain, relative to WT brains, as well as amelioration of behavioral deficits following transplantation of MSCs, we hypothesize that the observed benefits are not a result of immunomodulation.

Contrary to late-stage human HD, late-stage R6/2 mice do not have massive loss of striatal neurons. However, significant decreases are observed in the levels of metabolically active tissue within the striatum, which is suggestive of striatal dysfunction [29]. This study was able to demonstrate a neuroprotective effect of transplanted BM MSCs. These results are similar to what has been observed following MSC transplantation into the N171-82Q mouse model of HD [30], although few surviving transplanted cells were reported in this study. In QA lesion models, a reduction of striatal atrophy has been observed following MSC transplantation, with a small number of surviving cells observed at 16 weeks after transplantation [31, 32].

The ability of MSCs to survive following transplantation into the brain is currently being studied in several laboratories, with widely varying results being reported. In the current study, no significant difference in the number of surviving MSCs was found between high-passage and low-passage groups at 6 weeks following transplantation. This is similar to other studies that have observed robust MSC survival following transplantation in a QA rat [33, 34]. We have recently reported that transplantation of rat BM MSCs into the rat striatum decreases the general activity of the hypoimmunogenic dendritic cells, macrophages, and T lymphocytes, which translated into reducing the probability of MSC rejection [20]. Because cytogenic analyses were not performed on MSCs in the current study, the possibility that chromosomal abnormalities may have influenced the present results cannot be ruled out. However, despite findings that murine BM MSCs are highly susceptible to chromosomal abnormalities as early as 10 days in culture [19, 35, 36], tumor formation following MSC transplantation into the brains of rodent HD models has not been observed.

As the numbers of surviving BM MSCs in the present study were not different between the two different passage groups after transplantation, the differences observed in behavioral and histological sparing between mice receiving low-passaged or high-passaged MSCs were not due solely to the number of MSCs in the brain. Further, as BM MSCs were not seen to differentiate into neuronal-lineage cells, replacement of lost or damaged cells is not responsible for the behavioral and histological results. Other mechanisms such as secretion of trophic factors, like BDNF, therefore need to be identified to account for the therapeutic effects of the transplanted MSCs.

It has been found that patients with symptomatic HD with lower serum concentration levels of BDNF have significantly impaired motor and cognitive performances, relative to individuals with normal BDNF levels [37, 38]. It is also shown that BDNF is very important for survival and differentiation of striatal neurons, and that lower BDNF may be a causative factor in the deterioration of specific neuronal populations that is observed in HD [6].

In the current study, transplantation of BM MSCs in the striata of R6/2 mice significantly restored BDNF expression levels. As the loss of BNDF mRNA expression has been shown to be significantly reduced in 6-week-old R6/2 mice [22], we postulate that the secretion of BDNF from transplanted BM MSCs when R6/2 mice were 5 weeks of age prevented this age-dependent loss of BDNF mRNA expression in these R6/2 mice. Although we did not directly measure BDNF protein levels in this study, we have previously observed an increase in BDNF immunolabeling within the striatum following transplantation of MSCs [20], which supports our contention that BDNF upregulation may play a major role in the observed behavioral sparing in the present study.

Our findings confirm those of Jiang and colleagues, who observed an upregulation of BDNF following MSC transplantation in a toxic lesion model of HD [39]. The capacity of MSCs to secrete growth factors and other neuroprotective factors *in vitro* and *in vivo* following transplantation has been well documented [8, 40, 41]. However, based on the current evidence, we are unable to determine whether the BDNF is secreted from the transplanted cells, or whether it is upregulated in brain tissue as a result of BM MSC transplantation. Testing these hypotheses has become a focus of future studies in our laboratory.

While our study suggests that BDNF plays a critical role in the progression of HD symptoms and may be the main mechanism behind the behavior and neuropathological sparing, other neurotrophic factors are critical for disease progression as well. It has been shown that viral delivery of glial-derived neurotrophic factor can ameliorate accelerod deficits and reduce hind-limb clasping in the N171-Q82 mouse model of HD, similar to our findings in the R6/2 model [42]. Clearly, more work is needed to delineate the relative contribution of the sundry putative factors that may underlie the functional benefits exerted by the transplanted MSCs.

Our study suggests that high-passage mouse BM MSCs are more suitable for transplantation in the R6/2 model. It is important to note, however, that this is likely to be different for transplantation of rat or human MSCs [20, 43–46]. These conflicting results between mouse, rat, and human MSCs add to an increasing body of evidence that different populations of MSCs are inadvertently selected as a result of passaging. To enhance clinical translation, careful characterization prior to transplantation is necessary in order to isolate the population most beneficial for the intended therapy and to ensure safety against chromosomal instability.

## Conclusions

In summary, our study provides additional evidence that transplantation of BM MSCs holds significant promise to delay the onset of motor, cognitive, and neuropathological loss in HD, most probably through the release of neurotrophic factors, specifically BDNF. This study also underscores the need to consider the passage number when evaluating the efficacy of MSC transplantation therapies.

## Abbreviations

ANOVA: analysis of variance
BDNF: brain-derived neurotrophic factor
BM: bone marrow
CYO: cytochrome oxidase
GFAP: glial fibrillary acid protein
HD: Huntington’s disease
HSD: honestly significant difference
htt: huntingtin
MHC: major histocompatibility complex
MSC: mesenchymal stem cell
MWM: Morris water maze
NeuN: neuronal nuclei
NGF: nerve growth factor
PBS: phosphate-buffered saline
QA: quinolinic acid
TNF: tumor necrosis factor alpha
TrkB: tyrosine receptor kinase type 2
WT: wild type.
